# Comparison of Effectiveness and Safety of Oxycodone Hydrochloride and Fentanyl for Post-operative Pain Following Total Hip Arthroplasty: A Randomized Triple-Blind Trial

**DOI:** 10.5812/aapm-142710

**Published:** 2024-02-16

**Authors:** Neveen Kohaf, Salama A Harby, Ahmed F Abd-Ellatief, Mohamed A Elsaid, Neazy A Abdelmottaleb, Tamer F Abd Elsalam

**Affiliations:** 1 Lecturer of Clinical Pharmacy, Faculty of Pharmacy (Girls), Al-Azhar University, Cairo, Egypt; 2 Lecturer of Anesthesiology, Intensive Care and Pain Management, Damietta Faculty of Medicine, Al-Azhar University, Damietta, Egypt; 3 Assistant Professor of Anesthesiology, Intensive Care and Pain Management, Damietta Faculty of Medicine, Al-Azhar University, Damietta, Egypt

**Keywords:** Oxycodone Hydrochloride, Fentanyl, Total Hip Arthroplasty, Analgesia

## Abstract

**Background:**

Total hip replacement (THR) is frequently associated with intense post-surgical pain. Effective pain management is of crucial importance to improving patient's condition and increasing his/her satisfaction in the post-operative time.

**Objectives:**

This study aimed to compare the analgesic effect and safety of oxycodone and fentanyl after THR.

**Methods:**

Seventy-two cases scheduled for elective THR were included in this randomized, triple-blind trial. The patients were equally randomized into 2 groups: Fentanyl group (50 ug of fentanyl) and oxycodone group (oxycodone 4 mg). Drugs were received 20 min prior to the end of the operation.

**Results:**

Post-operative visual analog scale (VAS) measurements at rest and movement at the post-anesthesia care unit (PACU) and in the ward, 2 h, 4 h, and 8 h post-operatively exhibited a significantly reduced value in the oxycodone group compared to the fentanyl group (P-value < 0.05). Time to first rescue for analgesia was delayed significantly in the oxycodone compared to the fentanyl group (P-value < 0.001). Fentanyl consumption (ug) in the 1st post-operative 12 h, 24 h, and 48 h decreased significantly in the oxycodone group compared to the fentanyl group (P-value < 0.001). Post-operative nausea, vomiting, headache, and pruritus were matched between the 2 groups (P > 0.05).

**Conclusions:**

A bolus dose of 4 mg of oxycodone provided superior analgesic efficacy than 50 ug fentanyl as evidenced by significantly lower pain score, delayed onset to first request for analgesia, and the smaller amount of fentanyl consumption at 12, 24, and 48 h post-total hip arthroplasty compared to fentanyl. The incidence of adverse events was comparable between the 2 groups.

## 1. Background

Effective pain relief is of crucial importance to improve a patient's condition and increase his/her satisfaction in the time after surgery. Suboptimal pain management is usually associated with a prolonged duration of hospital stay and delayed rehabilitation, with a subsequent increase in the overall cost (1). In addition, inadequate control of pain after surgery could result in the development of post-surgical pain syndromes. Thus, effective pain control and relief are crucial, starting in the post-anesthesia care unit (PACU) and after a total hip replacement (THR) (2). 

Intravenous (IV) analgesia is often delivered in the primary post-operative episode, as oral administration is less practicable (3). In moderately severe to severe post-operative acute pain, guidelines advocate the use of potent opioids like morphine, fentanyl, oxycodone, or hydromorphone within the aspect of a multimodal analgesic strategy (4).

Fentanyl was the most commonly used opioid for post-operative analgesia as it has an action of rapid onset (5 - 7 min) (i.e., when rapid analgesia is needed within the recovery area or critical care unit). It is also associated with fewer requests for extra analgesics, a quicker recovery of the bowel, and a short duration of post-operative hospital stay compared to morphine (5). 

The semisynthetic opioid oxycodone hydrochloride is an agonist of μ and κ receptors, smooth muscles, and the central nervous system (CNS). Clinically, its main use is the management of acute and chronic discomfort (6). 

In the literature, oxycodone exhibited comparable or greater pain management than fentanyl and was recommended as an alternative to fentanyl for pain management post-operatively (7). Nevertheless, it was primarily used to treat visceral pain due to the kappa receptors' stimulation of analgesic effects (8, 9). A recent systematic review and meta-analysis by Raff et al. (10) concluded that oxycodone has more analgesic efficiency compared to fentanyl with fewer side effects, such as sedation, but the included studies were limited to abdominal surgeries. Kim et al. evaluated oxycodone and fentanyl for post-operative pain management in orthopedic surgery patients, but their study was limited by a small sample size, and they recommended generalization through large-scale research (11).

## 2. Objectives

This randomized trial was designed to examine the efficacy of oxycodone and fentanyl for post-operative pain management following THR.

## 3. Methods

This prospective randomized triple-blinded trial involved 72 cases of both sexes aged 28 to 60 years old, an American Society of Anesthesiologists (ASA) physical status classification of I and II, who were planned for elective THR. The study was performed from November 2022 to May 2023. The study was conducted at Al-Azhar University (Damietta) Hospitals.

Written informed consent was obtained from all patients. The research was performed after the approval of the Ethical Committee of Al-Azhar University (Damietta) Hospitals (approval code: IRB 00012367-22-010-005), registration of clinicaltrials.gov (ID: NCT05602519), the date of first registration: 28/10/2022).

Exclusion criteria were a history of medication allergies, drug addiction, mental or emotional concerns, and chronic kidney and liver impairment.

 

### 3.1. Randomization and Blindness 

Utilizing computer-generated randomization numbers, we randomly assigned the patients equally into 2 groups: The fentanyl group (n = 36), including patients who received 50 ug of fentanyl, and the oxycodone group (n = 36), including patients who received 4 mg of oxycodone. Sealed, opaque, sequentially numbered envelopes were used to ensure random allocation by a nurse who did not take part in the study. Drugs were received 20 min prior to the end of the operation. Patients, observers, and the outcome evaluators were unaware of the trial pharmaceuticals. Drugs were prepared by an additional pharmacist who did not join the remaining phases of the trial. All containers were sealed by aluminum foil, identical in appearance.

### 3.2. Preoperative 

Patient-specific histories were taken, and clinical examinations and regular laboratory investigations were performed. During the preoperative appointment, patients were instructed on the use of the visual analog scale (VAS) (0: No discomfort and no pain; 10: Extreme discomfort and maximal pain). An IV line and urinary catheter were inserted. Cases were connected to a monitor consisting of pulse oximetry, non-invasive blood pressure, 5-lead ECG (electrocardiogram), a temperature probe, and capnography. 

### 3.3. Intraoperative

Anesthesia was administered by a standard technique. It was induced by propofol IV (1 to 2 mg/kg), rocuronium (0.8 mg/kg), and fentanyl (1 µg/kg). Anesthesia was then maintained by isoflurane (1.2 %), delivered in a 50% combination of oxygen and air. Top-up doses of rocuronium and fentanyl were given as needed. Standard monitoring was performed during the surgery and consisted of ECG, blood pressure, oxygen saturation, end-tidal carbon dioxide (CO_2_), temperature, and peripheral nerve stimulator monitoring. 

A triple-blind administration occurred 20 minutes prior to the surgery termination; 50 ug of fentanyl was administered to the fentanyl group, and 4 mg of oxycodone was administered to the oxycodone group. 

Isoflurane was stopped at the end of the surgery. Sugammadex 2 mg/kg was given IV when spontaneous recovery was reached, with the reappearance of the second twitch in response to TOF (train of 4) to reverse muscle relaxation. 

After confirmation of adequate spontaneous respiration and sufficient consciousness recovery, extubation was performed. Then, patients were transferred to the PACU and put under observation for 30-45 minutes before they were transferred to the inpatient ward. 

When the VAS was > 4 in the PACU, a fentanyl bolus dose of 50 ug was administered to the patients as the rescue analgesia regardless of his/her assigned group. In addition, in the ward, a similar IV dose of fentanyl (as in PACU) was administered for severe pain (VAS score > 4). 

Mean arterial blood pressure (MBP) and heart rate (HR) were recorded at the baseline, PACU, 2 h, 4 h, 8 h, 12 h, 24 h, and 48 h post-operatively.

The VAS at rest and movement was recorded at PACU at 2 h, 4 h, 8 h, 12 h, 24 h, and 48 h post-operatively.

The time of fentanyl administration and the overall dose administrated were documented at 12, 24, and 48 h post-operatively. In addition, any adverse events were documented. Post-operative nausea and vomiting (PONV) were treated by IV administration of metoclopramide (10 mg). 

The primary outcome was improving the PACU pain score. The secondary outcomes were the timing of the initial request for analgesia, hemodynamics (HR, MBP), post-operative side effects, and post-operative consumption of opioids. 

### 3.4. Sample Size Calculation

The sample size determination was done by G*Power v. 3.1.9.2 (Universitat Kiel, Germany). The sample size was estimated as N ≥ 31 in each group based on the following considerations: .0941 effect size, 0.05 α error, and 95% power based on the data of a previous study (11), as the mean pain score (the primary outcome) was 4.96 ± 1.64 in the fentanyl group and 3.48 ± 1.5 in the oxycodone group. Five cases were added to each group to overcome dropouts. Therefore, 36 cases were enrolled in each group.

### 3.5. Statistical Analysis 

SPSS v. 28 (IBM2, Armonk, NY, USA) was employed to conduct statistical analysis. Histograms and the Shapiro-Wilks test were utilized to examine the normality of the data distribution. Quantitative parametric data were reported as mean and standard deviation (SD) and were evaluated using an unpaired Student's *t* test. Non-normal quantitative data were assessed using the Mann-Whitney U test and displayed as median and IQR (interquartile range). When applicable, the chi-square test or Fisher's exact test was used to analyze qualitative variables, provided as frequencies and percentages. When the P-value was less than 0.05 with 2 tails, it was judged significant.

## 4. Results

**Figure 1.**
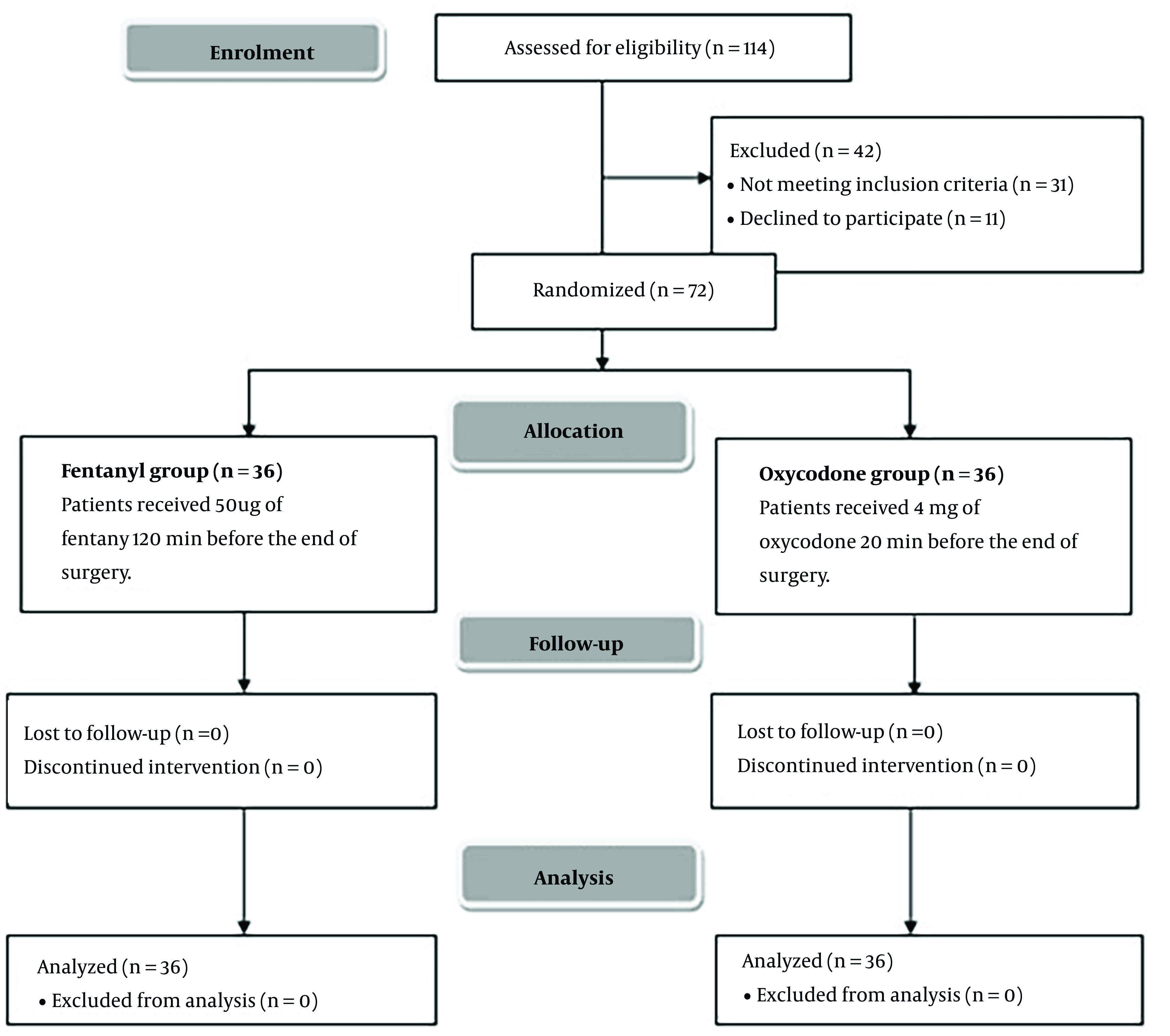
CONSORT flowchart of the enrolled patients

In this study, 114 patients were evaluated for suitability; 31 patients did not meet the criteria, and 11 patients declined participation in the study. The remaining patients were allocated randomly into 2 groups (36 per group). All the allocated patients were monitored and statistically analyzed (Figure 1). 

Patient characteristics and surgery duration were compared between the 2 groups (Table 1). 

**Table 1.** Patients' Characteristics and Duration of Surgery in the Studied Groups

Variables	Fentanyl Group (n = 36) ^a^	Oxycodone Group (n = 36) ^a^	P-Value
**Age (y)**	52.22 ± 6.07	53.56 ± 6.79	0.383
**Sex**			0.789
Male	26 (72.22)	27 (75)	
Female	10 (27.78)	9 (25)	
**Weight (kg)**	77.06 ± 9.34	77.47 ± 10.64	0.860
**Height (m)**	1.73 ± 0.06	1.72 ± 0.08	0.765
**BMI (kg/m** ^**2**^ **)**	25.73 ± 3.62	26.34 ± 4.93	0.551
**ASA physical status**			0.126
I	9 (25)	4 (11.11)	
II	27 (75)	32 (88.89)	
**Duration of surgery (min)**	159.92 ± 12.05	161.03 ± 9.95	0.671

Abbreviations: BMI, body mass index; ASA, American Society of Anesthesiologists.

^a^ Data are presented as mean ± SD or frequency (%).

**Figure 2.**
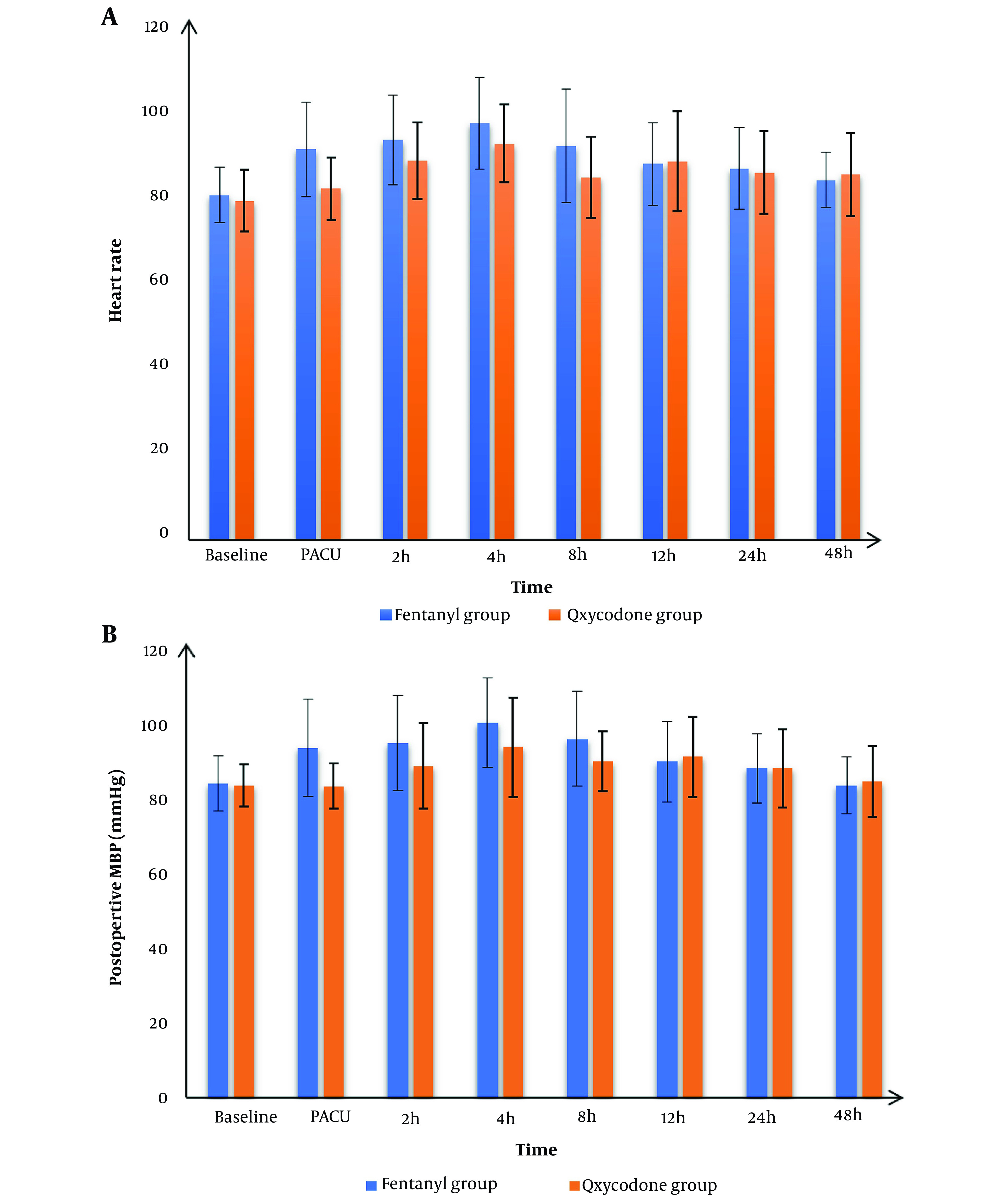
A, post-operative heart rate (HR), B, post-operative mean blood pressure (MBP) of the studied groups

Post-operative HR and MBP readings at baseline, 12 h, 24 h, and 48 h post-operatively were matched between the 2 groups and were significantly lower at PACU, 2 h, 4 h, and 8 h post-operatively in the oxycodone than fentanyl group (P-value < 0.05) (Figure 2). 

The VAS measurements at rest and at movement at PACU, 2 h, 4 h, and 8 h post-operatively were significantly lower in the oxycodone than in the fentanyl group (P-value < 0.05) and insignificantly differed between the 2 groups at 12 h, 24 h, and 48 h post-operatively (P-value > 0.05) (Table 2). 

**Table 2.** Visual Analog Scale Measurements of the Studied Groups

Variables	Fentanyl Group (n = 36) ^a^	Oxycodone Group (n = 36) ^a^	P-Value
**At rest**			
PACU	4 (3.75 - 5)	2 (1 - 2)	< 0.001 ^b^
2 h	4 (4 - 5)	3 (2 - 4)	< 0.001 ^b^
4 h	5 (4 - 5.25)	4 (3 - 5)	0.006 ^b^
8 h	4 (3 - 5)	2 (2 - 4)	< 0.001 ^b^
12 h	5 (4 - 5.25)	4 (4 - 5)	0.167
24 h	4 (4 - 5)	4 (2 - 4.25)	0.112
48 h	4 (3.75 - 4)	4 (2 - 4.25)	0.375
**At movement**			
PACU	5 (4 - 6)	2 (2 - 3)	< 0.001 ^b^
2 h	5.5 (4 - 6)	4 (3 - 5.25)	0.005 ^b^
4 h	6 (5 - 7)	5 (4 - 6.25)	0.037 ^b^
8 h	5 (4 - 6)	4 (3 - 5)	0.003 ^b^
12 h	6 (4 - 7)	5 (4 - 6)	0.079
24 h	5 (4 - 6)	5 (3.75 - 6)	0.102
48 h	4 (3 - 6)	4 (3 - 4.25)	0.166

Abbreviation: PACU, Post-anesthesia care unit.

^a^ Data are presented as median (interquartile range).

^b^ P-value ≤ 0.005 was considered statically significant.

Time to the first analgesic request was significantly longer in the oxycodone than in the fentanyl group (P-value < 0.001). The incidence of PONV was 6 (16.67%) in the fentanyl group and 10 (27.78%) in the oxycodone group. Six (16.67%) patients had headaches in the fentanyl group, while 9 (25%) exhibited headaches in the oxycodone group. There were 9 (25%) and 7 (19.44%) patients who had pruritus in the fentanyl and oxycodone groups, respectively. The PONV, headache, and pruritus were insignificantly different between the 2 groups (P > 0.05). No patient had respiratory depression in either group (Table 3). 

**Table 3.** Time to the First Rescue Analgesia and Incidence of Side Effects in the Studied Groups

Variables	Fentanyl Group (n = 36) ^a^	Oxycodone Group (n = 36) ^a^	P-Value
**Time to the first rescue analgesia (h)**	0.85 ± 0.49	4.44 ± 3.85	< 0.001 ^b^
**PONV**	6 (16.67)	10 (27.78)	0.257
**Headache**	6 (16.67)	9 (25)	0.384
**Pruritus**	9 (25)	7 (19.44)	0.571

Abbreviation: PONV, Post-operative nausea and vomiting.

^a^ Data are presented as mean ± SD or frequency (%).

^b^ Significant P-value ≤ 0.05.

**Figure 3.**
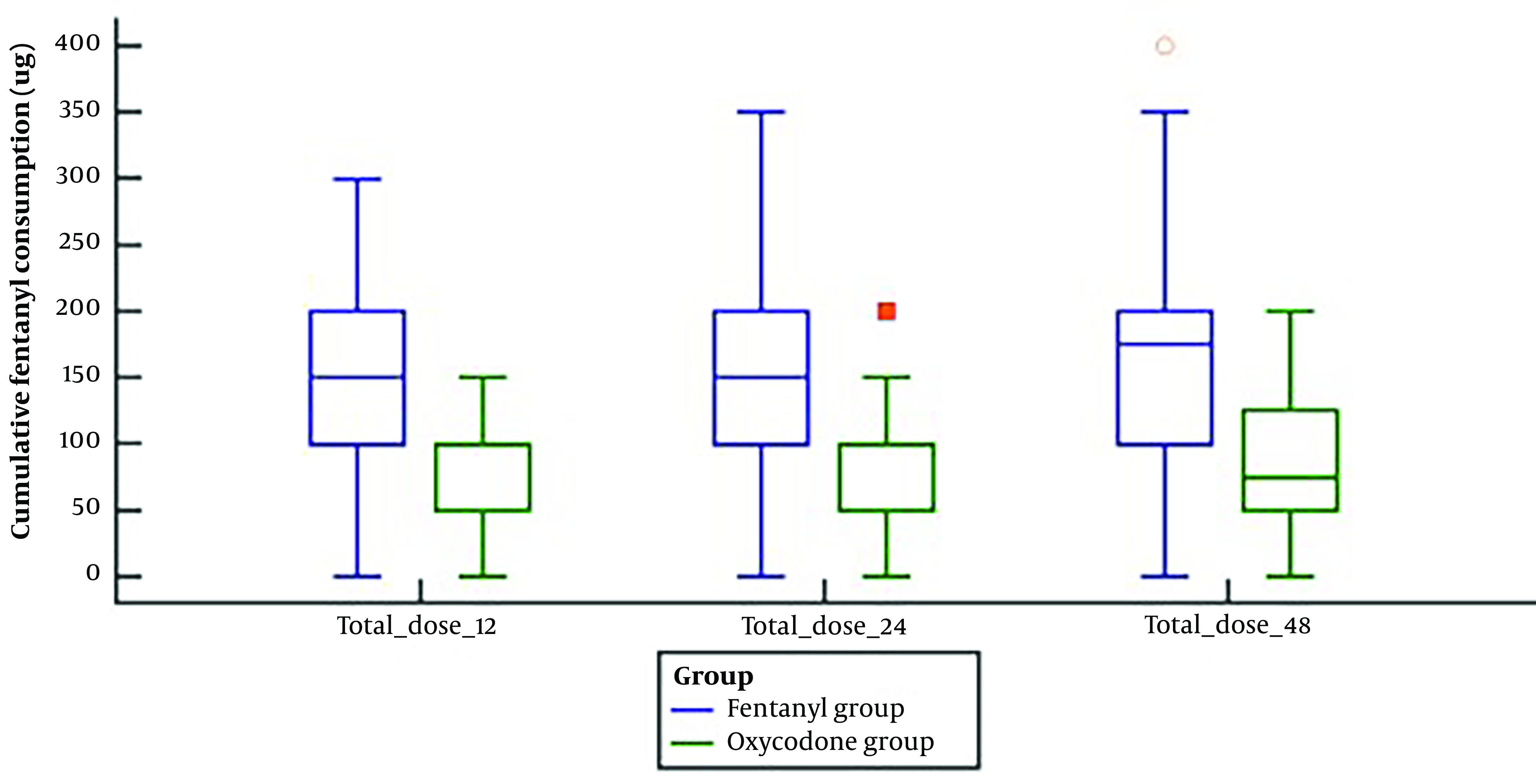
Cumulative fentanyl consumption of the studied groups

Fentanyl consumption (ug) in the 1st post-operative 12 h, 24 h, and 48 h was significantly lower in the oxycodone group than in the fentanyl group (P-value < 0.001) (Figure 3). 

## 5. Discussion

Adequate pain control post orthopedic surgeries is crucial to ensure early mobilization and a better quality of life (12). Opioids are the standard analgesics administered post-surgeries due to their strong analgesic effect (3). 

A dose of 50 mg fentanyl and 4 mg oxycodone was administered 20 minutes before the end of the surgery, guided by established potency ratios. Equivalent doses were necessary to assess the analgesic effects of these drugs. In a study following laparoscopic cholecystectomy, IV fentanyl (100 mg) was compared to oxycodone (10 mg), revealing lower pain scores for oxycodone but a higher incidence of side effects, particularly severe nausea and vomiting (13). This study was pivotal in establishing equivalent doses for oxycodone and fentanyl, with subsequent investigations exploring lower oxycodone doses. Intravenous oxycodone is recognized as having an equivalent or 3: 4 ratio to morphine. Comparisons with morphine and fentanyl suggested varying potency ratios, such as 1: 100 and 1: 75 for fentanyl to oxycodone (11). Given concerns about side effects, a ratio of 1: 80 was utilized in the mentioned study (13). 

Our results indicated that oxycodone induced analgesic effect superior to fentanyl, evidenced by significantly lower pain score at rest and at movements, lower HR and MBP, delayed onset to the first request for analgesia, and smaller amount of fentanyl consumption at 12, 24, and 48 h after total hip arthroplasty compared to fentanyl.

The powered analgesic efficacy of oxycodone could be attributed to the fact that the half-life of oxycodone is 3 to 6 h; however, comparable to our study, extended durations of activity than the half-life of oxycodone have been recorded (14). It has been observed that stimulation of the peripheral nervous system and the CNS may result in the progression of acute pain into chronic pain (15). The peripheral nervous system's sensitivity to pain increases the signal to the spinal cord, resulting in dorsal horn hyper-excitation and CNS sensitization. This progressively decreases the pain threshold and heightens the pain response. It is believed that oxycodone's efficient analgesic impact reduces pain sensitivity in the peripheral and CNS, resulting in a longer duration of action than the half-life (11).

Contrary to the majority of potent opioids, which work predominantly through the u opioid receptor to elicit an analgesic effect (16, 17), oxycodone exhibits extra-agonistic activity on the κ- and δ-opioid receptors; its further affinity to the κ -receptor is thought to be especially important for anti-nociception in the system of visceral pain (10). Furthermore, oxycodone has been reported to provide longer-lasting analgesic action than fentanyl (18).

Raff et al.'s (10) systematic review and meta-analysis included 6 studies with a total of 466 patients, comparing oxycodone with fentanyl. Supporting our findings, their net results were in favor of oxycodone in terms of analgesic power, as oxycodone exhibited lower pain score readings and significantly lower cumulative opioid consumption, with oxycodone compared to fentanyl between 8 and 48 h.

After THR surgery, Kim et al. (11) reported that the pain score reduced significantly in the PACU, and fewer patients in the PACU needed further fentanyl boluses in oxycodone compared to fentanyl. The accumulated need for opioids was lower significantly in also in oxycodone at 6, 12, 24, and 48 h post-operatively. 

Regarding safety results, our findings revealed that the incidence of PONV was 6 (16.67%) in the fentanyl group and 10 (27.78%) in the oxycodone group. Six (16.67%) patients had headaches in the fentanyl group, while 9 (25%) exhibited headaches in the oxycodone group. There were 9 (25%) and 7 (19.44%) patients with pruritus in the fentanyl and oxycodone groups, respectively. The PONV, headache, and pruritus were insignificantly different between the 2 groups.

In prior research, oxycodone was associated with a greater frequency of PONV than fentanyl (7, 19). The specific mechanism through which opioids generate PONV is unclear. Multiple opioid actions, such as augmentation of vestibular sensitivity, direct actions on the trigger zone of the chemoreceptor, and slowed gastric emptying, may be involved.

Vestibular sensitivity may result in dizziness, which may be brought on by the activation of mu-opioid receptors in the vestibular epithelium by opioids (20). 

Kim et al. (19) reported that the incidence of PONV was 31 (48.4%) and 8 (12.5%) in oxycodone with a significantly higher value than the fentanyl group.

The lower incidence of PONV in our study compared to Kim et al. may be because our patients received a single bolus dose of oxycodone, whereas other studies used continuous oxycodone injection via PCA that increased the frequency of adverse effects.

However, in contrast to our findings, Hwang et al. (18) observed no vomiting with any therapy.

Regarding pruritus, Hwang et al. (18) documented that the incidence of pruritus was lower with oxycodone (2.4 %) than with fentanyl (7.5 %), although this difference was statistically insignificant. Kim et al. (19) and Kim et al. (21) noticed a higher incidence of pruritus with oxycodone than with fentanyl (14 % vs. 4.8%) and (13.3 vs. 10.0%), respectively; however, these differences were statistically insignificant. 

The trial's short follow-up and single-center design are its significant limitations. Thus, future large-scale multicenter collaborative research and longer monitoring duration are warranted in order to confirm our results.

### 5.1. Conclusions

In conclusion: A bolus dose of 4 mg of oxycodone provided superior analgesic efficacy than 50 ug of fentanyl, as evidenced by significantly lower pain score at rest and at movement, delayed onset to the first request for analgesia, and a smaller amount of fentanyl consumption at 12, 24, and 48 h after total hip arthroplasty compared to fentanyl. The incidence of PONV, headache, and pruritus were comparable between the 2 groups.

## Clinical Trial Registration Code:

Registration of clinicaltrials.gov (ID: NCT05602519) and the date of first registration was (28/10/2022).
